# Functional Neurology and Photobiomodulation as Alternative Non-pharmacologic Treatment Modalities for Chronic Pain: A Narrative Review

**DOI:** 10.7759/cureus.110293

**Published:** 2026-06-05

**Authors:** Rebecca A Praetzel, Alexander Badman, Charles Joseph

**Affiliations:** 1 Osteopathic Manipulative Medicine, Liberty University College of Osteopathic Medicine, Lynchburg, USA; 2 Neurology, Liberty University College of Osteopathic Medicine, Lynchburg, USA

**Keywords:** alternative medical therapies, chronic pain management, functional neurology, low-level laser therapy, non-pharmacologic treatment, photobiomodulation

## Abstract

Chronic pain affects many adults worldwide. Its prevalence increases with age, making it an important and growing public health concern. At the neurophysiological level, pain transmission follows a complex neuronal pathway. With repeated activation, this system can become sensitized, contributing to the persistence of chronic pain through central augmentation. Given its complexity, chronic pain has been approached through multiple medical paradigms, including allopathic/pharmaceutical, osteopathic, chiropractic, and psychological therapies. More recently, functional neurology has emerged as a noninvasive therapeutic approach. This field operates on the principle that stimulation of intact neural circuits can influence and rehabilitate neighboring dysfunctional neurons, thereby improving overall neural function. Another treatment modality for chronic pain is photobiomodulation, also known as low-level laser therapy (LLLT). This technique has gained attention for its application across a myriad of conditions and shows promise in the treatment of chronic pain. Because human tissues absorb specific wavelengths of light differently, targeted application can optimize therapeutic effects. Using these principles, LLLT has been successfully applied in the management of several chronic pain conditions, including carpal tunnel syndrome, lateral epicondylitis, nonspecific low back pain, and fibromyalgia. Our review found that LLLT intervention led to statistically significant improvement in chronic pain patients affected by osteoarthritis, carpal tunnel syndrome, epicondylitis, low back pain, and fibromyalgia. This review aims to highlight the potential of functional neurology as an innovation to the approach to chronic pain management, with particular emphasis on the emerging role of photobiomodulation. It seeks to encourage further research into these modalities as potentially transformative options for patient care.

## Introduction and background

Chronic pain is a pathologic experience, arising from either inflammation and the subsequent and recurrent nociceptive signaling and/or from rewiring of the nervous system [1]. It is pain that persists longer than what is considered the normal timeframe of healing [2], usually defined as lasting over three months. The International Classification of Diseases categorizes it based on etiology as either chronic primary pain, which has no known cause and would include “conditions such as non-specific low back pain and fibromyalgia” [3], or chronic secondary pain, which arises secondary to another condition such as cancer, musculoskeletal dysfunction, or surgery.

Chronic pain is a widespread condition in the United States. Based on 2023 data from the CDC’s National Center for Health Sciences, an estimated 24.3% of the adult population is burdened by this disease state [4]. The prevalence tends to increase with age, with about 36% of individuals 65 years of age and older experiencing it. For an estimated 8.5% of adults, their condition interferes with activities of work or daily living, deeming it high-impact chronic pain. Dealing with chronic pain has an economic burden on individuals and the healthcare system as a whole. While pharmacologic interventions have great success for many people, for others, their effects are not sufficient; one treatment modality may be successful for many with a particular type of chronic pain while not for others with a different pain classification, or even those under the same category. Other times, there are more effective modalities with fewer side effects available, but they are underutilized. 

Numerous studies have reviewed a variety of non-invasive non-pharmacologic therapies for chronic pain and their efficacy. Techniques including osteopathic manipulations, chiropractic adjustments, massage therapy, acupuncture, tai chi, transcutaneous electrical nerve stimulation, cryotherapy, and heat therapy have shown benefit in many patients [5,6]. Additionally, due to the psychological implications of chronic pain, cognitive behavioral therapy, mindfulness-based therapy, emotional awareness and expression therapy, acceptance and commitment therapy, pain reprocessing therapy, music therapy, and even virtual reality therapy have been shown to be successful in reducing the burden of chronic pain. 

Often, a combination of the aforementioned interventions provides relief, but some individuals still seek further help. With an increase in desire for non-pharmacologic management of chronic conditions and of chronic pain in particular [7], knowledge of further alternative measures will prove beneficial. The goal of this review is to explore less widely known alternative treatment modalities for chronic pain, to expand knowledge on these interventions, and to encourage further research into potentially life-changing care, with a focus on functional neurology and photobiomodulation.

Physiology of chronic pain 

**Figure 1:**
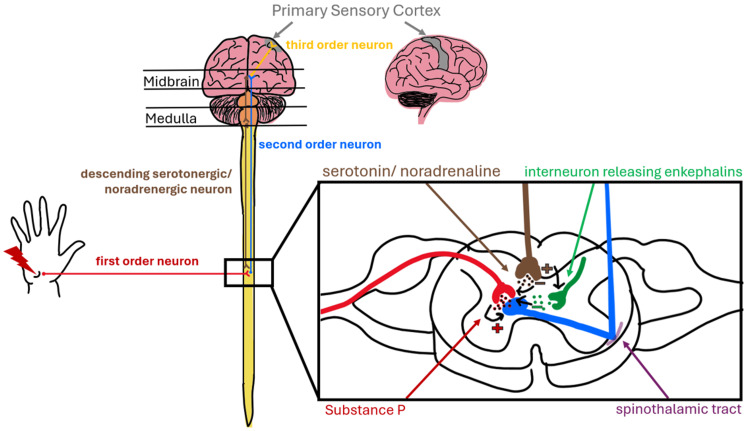
The Pain Pathway Simplified Image credits: Developed by the authors using Microsoft PowerPoint (Microsoft, Redmond, WA, USA).

In simplified terms, in healthy functioning pain pathways, nociceptors detect a noxious stimulus in the periphery. This first-order neuron releases substance P to trigger a second-order neuron. This second-order neuron ascends within the spinothalamic tract of the spinal cord to synapse on the third-order neuron within the cerebral cortex [8]. The descending pathway then begins with first-order neurons in the periaqueductal gray matter of the midbrain that descend to synapse on second-order, serotonergic/noradrenergic neurons in the nucleus raphe magnus of the medulla. This neuron synapses in the same area of the dorsal horn of the spinal cord that the first- and second-order neurons of the ascending pathway synapse, the substantia gelatinosa. Its release of serotonin and norepinephrine leads to inhibition of the release of substance P and the subsequent pain signaling to the brain. There are also interneurons in the substantia gelatinosa, which are activated by the release of serotonin and norepinephrine. Interneurons release enkephalins, which act on the opioid receptors to inhibit further release of substance P and the persistent perception of pain that would have followed [8]. This pathway is illustrated in Figure 1.

The abnormalities in chronic pain arise from various mechanisms. Nociceptors can be hypersensitive to any stimulus, or they can be improperly activated by stimuli that normally would not be activating at all [5]. In the brainstem of those with chronic pain, there is evidence of increased activating signals within neurons of the dorsal horn and spinothalamic tract. Within the brain, “prolonged nociceptive signaling leads to central sensitization, further amplifying pain signaling” [5], and decreased connections between the anterior cingulate cortex and the periaqueductal gray matter. The result is reduced inhibition of pain and, therefore, cognitive and emotional perception of continual pain. Disinhibition in the brainstem and within the descending pathway is downregulated as well. If the nervous system can be changed, regulated, and normalized by non-pharmacologic mechanisms, chronic pain may decrease to a level where drugs are not necessary.

Functional neurology and photobiomodulation 

Functional neurology is a field of medicine that seeks to solve health impairments by leveraging the neuroplasticity of the nervous system. Practitioners include physicians and chiropractors who have completed fellowships or certification of extra training in this field. It involves the incorporation of “sensory stimulation, motor activities, cognitive tasks, and emotional events” [9] to access regions of the brain in proximity to each other; by stimulating a region of the brain adjacent to the area responsible for a dysfunction, functionality should be augmented when that region of the brain is activated.

Photobiomodulation, also known as low-level laser therapy (LLLT), has its origins in the late 19th century, under the work of Niels Ryberg Finsen [10]. His work with light therapy had success in treating skin disruptions, from smallpox with red light in 1893 to lupus in 1895, earning him the Nobel Prize in Physiology or Medicine in 1903. His research is the groundwork for its application today. It has many further reports of treating dermatological conditions, including rosacea and acne, as well as playing a role in asthma, weight loss, hypothyroidism, yeast infections, and fertility/conception [11]. It has been especially popular in relation to cosmetics and beauty. Colloquial and anecdotal evidence has been on the rise in recent times, hence the need for research on the mechanism, the safety, and the actual efficacy of red light therapy and its role in modern medicine.

## Review

Methods

This narrative review included articles accessed using the PubMed database and Google Scholar search engine. Keywords used in the searches included low-level laser therapy, LLLT, photobiomodulation, red light, chronic pain, and functional neurology. Open-access, peer-reviewed studies and reviews were included.

Functional neurology

**Figure 2:**
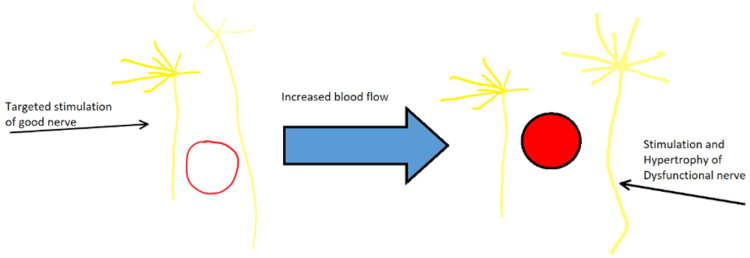
The Hypertrophic Principle of Functional Neurology Image credits: Developed by the authors using Microsoft Word (Microsoft, Redmond, WA, USA) and Microsoft OneNote (Microsoft, Redmond, WA, USA).

Functional neurology combines necessary lifestyle changes in regard to sleep, exercise, nutrition, cognition, and mental health to optimize its effectiveness and the overall health of the individual. The goal is to interpret pain signals correctly with restored, normal nervous system functioning and daily choices that optimize health; hence, there would no longer be a need to manage the chronic condition, as it would be treated [12]. There are a variety of neuromodulation treatment methods that are implemented to help with different forms of chronic pain. Their mechanisms involve stimulating a very specific region of the brain, brainstem, spinal cord, or nerve, which in turn regulates the improper nervous circuitry. Increasing blood flow to a nerve also increases supply to an adjacent nerve, as illustrated in Figure 2. Methods including but not limited to “repetitive transcranial magnetic stimulation (rTMS), transcranial direct current stimulation (tDCS), and transcranial alternating current stimulation (tACS)” deliver magnetic impulses or electrical currents to the brain in a non-invasive manner [13]. 

One method, tongue and facial nerve stimulation, balances autonomic tone and can regulate the pain pathway by the use of neurostimulation tools that administer vibration to the tongue or to the jaw around the temporomandibular joint [12]. The tongue has a multitude of nervous connections within the brainstem [14], an area crucial in initiating the descending pain pathway, and stimulation seeks to reverse the maladaptive changes that have occurred in this area. Eye and balance exercises are frequently utilized to alter pain and “reduce sensory overload” [12]. Restoration is accomplished via eye tracking and rebalancing the vestibular system. Activation of the vagus nerve is another commonly performed technique, used to tame the overactive pain signals as well as to decrease inflammation, and it can be done with rTMS, tDCS, or tACS. Moreover, techniques that activate the vagus nerve to normalize the parasympathetic tone have shown success. Techniques in that regard include cold therapy, breathwork, and light stimulation. Of light stimulation methods, photobiomodulation, most commonly in the form of red light therapy, will be further explored.

Overall, functional neurology provides a holistic, non-invasive approach to managing chronic pain that seeks to address the root cause. It may be of particular interest to osteopathic physicians because it aligns with osteopathic philosophy, especially the tenet stating that the body is capable of self-healing and self-regulation and that rational treatment is based on such [15]. Further research should study the mechanisms, proper methodologies, and efficacy of these treatments in hopes of providing further non-pharmaceutical options for safe and effective management of chronic pain.

Photobiomodulation 

This modality utilizes red light wavelengths of 620-700 nm and near-infrared wavelengths of 700-1440 nm in lasers or light-emitting-diodes (LEDs) directed into tissues to regulate cellular metabolism [16]. Hemoglobin and melanin within the skin absorb most light with wavelengths less than 600 nm, so higher ranges are needed for further penetration [17]. Superficial tissue is best treated with wavelengths of 600-700 nm, while deeper tissues need wavelengths typically between 780 and 950 nm. Interestingly, the 700-770 nm range is rarely used due to its failure to elicit significant biochemical activity. 

The chromophores within cells of the tissues at various depths then absorb a majority of the light to which they are exposed. A redox reaction then occurs; the photon absorbed causes electron excitability, resulting in electrons jumping to an outer orbit of higher energy. More energy is then available for cellular processes and healing. Evidence points to chromophores within mitochondria being the primary area of effect, supported by the resulting increase in mitochondrial products, including “ATP, NADH, protein, and RNA, as well as a reciprocal augmentation in oxygen consumption. Various in vitro experiments have confirmed that cellular respiration is upregulated” [17].

LLLT has additionally demonstrated downregulation of reactive oxygen species levels that would otherwise promote oxidative stress, modulation of levels of nitric oxide and calcium, and vasodilation [16]. It has been shown to cause upregulation of various transcription factors, including "redox factor-1 (Ref-1)-dependent activator protein-1 (AP-1) (a heterodimer of c-Fos and c-Jun), nuclear factor kappa B (NF-κB), p53, activating transcription factor/cAMP-response element-binding protein (ATF/CREB), hypoxia-inducible factor (HIF)-1, and HIF-like factor. These transcription factors then cause protein synthesis that triggers further effects downstream, such as increased cell proliferation and migration, modulation in the levels of cytokines, growth factors, and inflammatory mediators, and increased tissue oxygenation" [17].

As a result of those chemical changes, inflammation is combatted, wound healing is supported, and pain and other neurologic disorders are modulated. Despite what is known, much of the mechanism of LLLT has not been explored and remains unknown. Hence, its therapeutic use is considered controversial by some.

Optimization of this therapy relies on the aforementioned wavelengths in addition to the light energy per unit surface area of the skin termed the power density (mW/cm^2^), the length of treatment, the distance of the light from the skin or area of treatment, the pulse mode of the light being delivered, the diameter of the light on the skin surface termed the spot size, the amount of energy delivered per unit of skin surface termed the fluence (J/cm^2^), and the frequency of treatments [16]. The physics involved has been studied to elicit ranges for the parameters that are most effective at causing positive changes at the cellular level. However, specific values to be optimally efficacious have not been well studied. Certain ranges beneficial for one treatment show ineffective in another. Many studies on the efficacy of LLLT even yielded negative results, likely because the parameters chosen did not reach a therapeutic threshold [17]. While more research needs to be done on the topic, the World Association of Laser Therapy provides dosage recommendations based on the available evidence, empirical data, and extrapolation [18]. 

Numerous studies have been conducted on the effects of LLLT on different classifications of both acute and chronic pain. While photobiomodulation has frequently demonstrated success alone, it has been a promising therapy when used in combination with other relieving practices as well. One such example is the reduction of pain when combined with ultrasound in patients with knee osteoarthritis [19]. LLLT can deactivate trigger points when irradiating the triggering tendons or bursae. Similarly, when lasers with small spot points are applied with low power, pain relief has been attained in a manner similar to that of traditional Chinese acupuncture, which has been well studied in the context of chronic pain [20]. 

LLLT has led to statistically significant improvement in chronic neck pain 22 weeks after the conclusion of the therapy, as well as immediate pain relief following the treatment in 820 patients from 16 randomized clinical trials [21]. Osteoarthritis, patellofemoral pain syndrome, and mechanical spine disorders are among chronic joint disorders that have had successful pain amelioration, documented in at least 88 randomized controlled trials [20]. As previously discussed, the literature has data that are not all supportive of LLLT. While in some cases the negative results may be due to a true lack of benefit, other instances may be attributed to the use of improper parameters. The following sections will explore some specific classifications of chronic pain with various studies that support the benefits of photobiomodulation. 

Carpal tunnel syndrome

Chronic pain of carpal tunnel syndrome is a condition that has been tested with LLLT with interesting results. In a study of 60 patients treated for three weeks, there was a significant decrease in pain across all groups, treatment and placebo alike [22]. The treatment group received LLLT five days a week with a wavelength of 830 nm, a power density of 50 mW, and a fluence of 1.2 J/point. From this study, it cannot be determined that the therapy was ineffective, nor can it support the efficacy of the treatment modality. However, in a similar study but with different parameters, both chronic pain and functional status improved in 75 patients with a statistically significant difference from that placebo [23]. While these patients were also treated five times a week with wavelengths of 830 nm, the power density and fluence were both higher at 60 mW and 9.7 J/cm, respectively. With these data, it seems appropriate to conclude that this therapy yields positive results for patients struggling with carpal tunnel syndrome.

Lateral epicondylitis 

In a study of 50 elbows with lateral epicondylitis who received LLLT for three weeks with the parameters published by the World Association for Photobiomodulation Therapy, pain was assessed using the “visual analog scale, tenderness, Disability of the Arm Shoulder and Hand (DASH) questionnaire, the Patient-Related Lateral Epicondylitis Evaluation (PRTEE) test, pain-free grip strength, and the Nottingham Health Profile (NHP) questionnaire” [24]. There was no success in pain reduction acutely, immediately at the end of the three weeks of treatment, in either group. However, when the patients were reassessed three months following the completion of treatment, those who had received the laser therapy had significantly reduced pain compared to the placebo. 

In a randomized control trial including 39 patients who received LLLT along with a prescribed exercise routine for three weeks, pain relief was achieved along with improved grip strength [25]. A multicenter clinical study including 324 patients treated groups of patients from two to five weeks and found that combination techniques were most effective at reducing both acute and chronic pain and functionality for patients experiencing medial or lateral epicondylitis, as measured by visual analog scales, verbal rating scales, the patients’ pain diaries, hand dynamometer, and McGill's Pain Questionnaire [26]. The researchers here also emphasized the need for proper parameters, as they found that this therapy can be ineffective if under-radiated or even occasionally yield negative effects if over-radiated. 

Non-specific low back pain

The low back is a common area of chronic pain for various reasons, and photobiomodulation has again proven beneficial to those suffering from this condition. One study of 18 patients with non-specific low back pain quantified the presence of inflammatory markers alongside pain intensity levels after a single treatment with LLLT. Comparing 15 minutes before treatment to that after treatment, both pain and prostaglandin E2 levels were decreased. There was no significant difference in levels of necrosis factor-α or interleukin-6, suggesting that alteration of prostaglandin E2 levels may play an important role in the analgesic effects of LLLT in these patients [27]. 

Furthermore, in a randomized controlled trial of 40 participants with low back pain, statistical significance was found in the treatment of long-term pain; 89.5% of those receiving true laser therapy had no pain three months post-treatment, while 73.3% of those who received the sham laser placebo still reported pain at this time [28]. The laser was directed at multiple sites -- “articular spaces of vertebral column, adjacent paravertebral points, pain radiating areas, tender points, and also pain-controlling acupuncture points” [28] -- to achieve this result. Additionally, functional status and spinal range of motion showed significant improvement in the treatment group, clearly illustrating the osteopathic principle that “structure and function are reciprocally interrelated” [13]. Understanding these findings supports the claim that this non-invasive intervention may cause positive changes at the level of the dysfunction contributing to chronic pain, rather than by merely managing the symptoms. 

Fibromyalgia

The National Fibromyalgia Association estimates that 10 million people within the United States suffer from this diagnosis, and many find it difficult to manage [29]. Success often requires multimodal management. One randomized controlled trial evaluated the combination of LLLT and exercise training for its efficacy in fibromyalgia pain management of 160 women [30]. In this study, LED light therapy was applied to 11 different locations of the body. Pain was assessed using algometry, a quantification of the amount of pressure administered during palpation measured in kg/cm², and the visual analog scale. An immediate benefit was found in those receiving photobiomodulation therapy, with no additional advantage in the acute setting for those receiving the combination of that and exercise. Looking longer term at 10 weeks, both the LLLT and the combination group saw a significant decrease in pain and an improved quality of life as compared to the group receiving exercise alone and to the placebo group, but the combined therapy did yield a more substantial result [30]. 

One randomized clinical trial assessed the efficacy of photobiomodulation as compared to that of lidocaine 2% injections in the context of fibromyalgic orofacial pain in 66 adults [31]. LLLT was found to be as efficacious as lidocaine, as both led to a reduction of pain (p = 0.0001) with no statistical difference between the two groups. While this study examined a single treatment rather than a combination, this non-pharmacologic intervention alone helped as much as a medication. Both studies support the role of photobiomodulation in the management of a complex chronic pain disorder that is often difficult to treat.

## Conclusions

Chronic pain can be a challenge to treat, whether with medications or through a non-pharmacologic route. With an increased societal desire to seek more natural solutions to medical problems, alternative modalities that have been scientifically backed may provide beneficial solutions with minimal to no adverse effects. Functional neurology attends to the root cause of chronic pain, assisting the brain in rewiring back to a normalized state. The techniques utilized are non-invasive and without side effects. There is a need for randomized trials to determine an effect size and elicit quantifiable results. At a minimum, LLLT has proven to be an effective adjunct therapy in the treatment of chronic pain, and evidence exists for its utility alone as well. Some of the most common types of chronic pain, including osteoarthritis, carpal tunnel syndrome, epicondylitis, low back pain, and fibromyalgia, have been successfully improved with red light photobiomodulation. According to the evidence cited in this review, LLLT and functional neurology have significantly improved chronic pain in over 1,000 patients. Research should continue in these fields to augment the literature on these topics. Making effective management options more known, accessible, and commonplace can help millions of people struggling with chronic pain to live more normalized, authentically healthy lifestyles.
